# Transmission of Bacterial Endophytes

**DOI:** 10.3390/microorganisms5040070

**Published:** 2017-11-10

**Authors:** Anna Carolin Frank, Jessica Paola Saldierna Guzmán, Jackie E. Shay

**Affiliations:** School of Natural Sciences, University of California Merced, Merced, CA 95340, USA; jsaldiernaguzman@ucmerced.edu (J.P.S.G.); jshay@ucmerced.edu (J.E.S.)

**Keywords:** bacterial endophytes, transmission, vertical, horizontal, colonization, dispersion

## Abstract

Plants are hosts to complex communities of endophytic bacteria that colonize the interior of both below- and aboveground tissues. Bacteria living inside plant tissues as endophytes can be horizontally acquired from the environment with each new generation, or vertically transmitted from generation to generation via seed. A better understanding of bacterial endophyte transmission routes and modes will benefit studies of plant–endophyte interactions in both agricultural and natural ecosystems. In this review, we provide an overview of the transmission routes that bacteria can take to colonize plants, including vertically via seeds and pollen, and horizontally via soil, atmosphere, and insects. We discuss both well-documented and understudied transmission routes, and identify gaps in our knowledge on how bacteria reach the inside of plants. Where little knowledge is available on endophytes, we draw from studies on bacterial plant pathogens to discuss potential transmission routes. Colonization of roots from soil is the best studied transmission route, and probably the most important, although more studies of transmission to aerial parts and stomatal colonization are needed, as are studies that conclusively confirm vertical transfer. While vertical transfer of bacterial endophytes likely occurs, obligate and strictly vertically transferred symbioses with bacteria are probably unusual in plants. Instead, plants appear to benefit from the ability to respond to a changing environment by acquiring its endophytic microbiome anew with each generation, and over the lifetime of individuals.

## 1. Introduction

Plants are home to a myriad of microbes that live on below- and above-ground plant surfaces, called rhizosphere and phyllosphere, respectively. In addition, the last decade has witnessed an increased focus on endophytes, which are microbes that colonize the interior of plants without causing disease [1]. This is a broad and sometimes contested definition, as theoretically, the microbiome within an apparently healthy plant could consist of a mix of mutualistic, commensal, and latent pathogenic strains [2].

The plant microbiome is currently attracting a lot of research interest due to its ability to buffer plant hosts against abiotic and biotic stress, facilitate nutrient uptake and nutrient use efficiency, and promote growth [2,3,4,5,6,7,8,9,10,11,12]. Endophytic bacteria can be used to improve plant productivity and stress tolerance in the absence of pesticides and inorganic fertilizers, and to facilitate phytoremediation of heavy metals and hydrocarbons, but more research is needed on how to best inoculate plants in field settings [13]. Likewise, bacterial endophytes in wild plants play important roles in biotic and abiotic stress protection and nutrient acquisition [14,15,16,17,18], but in order to understand the significance of those processes at both the individual plant and ecosystem levels, we need a better understanding of endophyte colonization routes and dispersal modes. For example, to understand how much an individual forest tree may benefit from endophytic nitrogen fixation [16], or to estimate how much nitrogen is brought into a forest ecosystem via this pathway [15], we need to know when and how endophytic communities assemble. Here, we review the known and potential routes of transfer and dispersal of bacterial endophytes, and identify gaps in our understanding of how bacteria move among hosts, and between the host and the surrounding environment.

Host-associated microbes can colonize the host horizontally via the environment, vertically from within the parent to the offspring, or by mixed modes [19]. In many vertically transmitted symbioses, the symbiont is obligate and spends its entire life cycle inside the host, unable to survive in the environment [19]. Ecological and evolutionary relationships impact transmission mode and vice versa: Theory predicts that vertical transmission evolves when symbiotic partners are mutualistic, as a way to ensure faithful transmission of the beneficial symbiont from one generation to the next [20]. Vertical transmission of bacterial symbionts from parent to offspring is, indeed, common in systems where the symbiont provides an indispensable function, as in the extensively studied nutritional symbioses between bacteria and insects [21]. Vertical transmission via seeds is well documented for certain groups of fungal endophytes (e.g., the well-studied *Epichloë* and *Neotyphodium* fungal endophytes of grasses [22]). However, not all mutualists are obligate, and there are many examples of mutualistic horizontally transmitted symbioses [23,24].

Most bacterial endophytes are likely to be horizontally transmitted. First, the diversity of bacteria in seeds and seedlings raised under sterile conditions is typically lower than the diversity in plants grown in soil [25], suggesting that a majority of endophytes are acquired from the environment. Second, bacterial endophytes are often generalists, as beneficial properties of endophytes can typically transfer to distantly related plants [9,26,27]. Bacterial generalists that infect many different species of plants must move horizontally among them, and are unlikely to be strictly vertically transmitted.

Horizontal transmission of beneficial bacteria may be in the plant’s best interest. The ability to recruit a diverse set of symbionts from the environment may be advantageous for sessile organisms like plants, providing a means to respond to a changing environment [28]. Indeed, plants appear capable of hosting a large diversity of generalist endophytes whose presence or absence at a particular time depends much more on the plant’s environment (e.g., soil type) than its genotype [29,30,31,32,33,34]. Stronger host effects have been found when comparing more distantly related plant taxa, but even then, host genotype is less important than soil type [35].

In contrast, obligate relationships between bacteria and plants appear rare. It is possible that some bacterial endophytes are transmitted both vertically and horizontally (i.e., mixed-mode transmission), and an endophyte that is beneficial to its host under a particular circumstance (e.g., biotic stress) may be passed down to the offspring through the seed.

**Figure 1:**
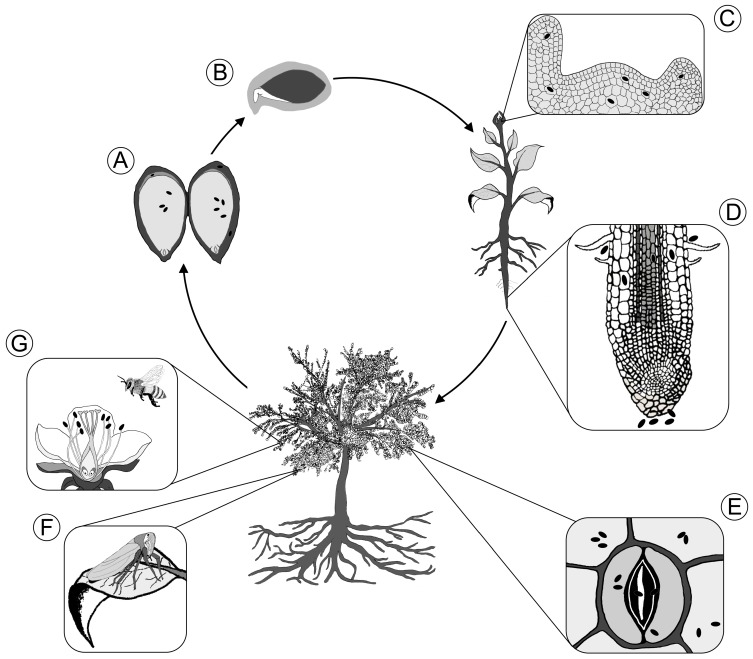
Transmission routes for bacterial endophytes across the life cycle of an apple tree. (**A**) Vertical transmission via seed; (**B**) Colonization of the spermosphere, depicted as the grey area surrounding the seed; (**C**) Colonization of developing reproductive organs via the shoot apical meristem as part of vertical transmission; (**D**) Colonization of root from soil; (**E**) Colonization of leaves though stomata after transmission via air; (**F**) Transmission via sap-feeders; (**G**) Transmission to flowers via pollinators. Not drawn to scale.

Here, we review known and potential routes of transmission for bacterial endophytes. The review is organized by transmission route (vertical vs. horizontal), rather than plant organ or order of importance. We begin by reviewing vertical transmission from the parent plant via seed or pollen, then move to horizontal transmission from the environment, starting with the germination environment and ending with the floral organs. Figure 1 summarizes the different transmission routes and modes reviewed. Vertical transmission of bacterial via seed and pollen likely occurs, as bacteria have been identified inside the seed of many different plant species (Figure 1A). Endophytes that are consistently transferred across generations must have a route from seeds to reproductive organs, either via xylem vessels or via the shoot apical meristem that differentiates into reproductive organs (Figure 1C). Soil is considered the dominant environment from which bacterial endophytes originate [36], and soil-to-root is the best-studied horizontal transmission route. Soil bacteria can colonize the plant interior and become endophytes early, via the germination environment called the spermosphere (depicted in grey in Figure 1B), or later through the rhizosphere and into the root of seedlings and adults (Figure 1D). The above-ground plant surface, or phyllosphere, is colonized with a diverse community of microorganisms, presenting an alternative, but less-studied, route of entry for bacteria that originate from rainwater, bioaerosols from surrounding soil, or from dust and other particles in the atmosphere, and potentially gain entry via stomata (Figure 1E). It is probable that stomata serve as a transmission route for plants of all life stages, but may be especially important for foliar endophytes of trees. In addition, sap-feeders and pollinators, and other arthropods may serve as vectors for bacteria that colonize the inside of plants (Figure 1F,G). Mixed transmission modes are probably not uncommon since, for example, bacteria applied to flowers can be transferred to the next generation [37]. However, the relative importance of different transmission routes is not known for most plants. To complicate matters more, many endo- and epiphytic bacteria can colonize plants above and below, inside and out [38,39], highlighting the importance of bacterial movement within the plant for the transmission of bacteria between plants. Bacteria have been shown to colonize the xylem vessels [40], which are thought to represent the main transport route for systemic colonization of interior plant tissues, though the process can take several weeks [26].

## 2. Vertical Transmission

### 2.1. Vertical Transfer via Seeds

The seed microbiome is increasingly attracting interest, and has been the subject of several recent reviews [7,41,42,43]. Bacteria have been detected in surface-sterilized seeds of various species, including crop plants like alfalfa [44], rice [25,45,46,47,48,49], maize [50,51], tobacco [52], coffee [53], quinoa [54], common bean [55], grapevine [56], barley [57], and pumpkin [58], but also in wild plants like the giant cardon cactus (*Pachycereus pringlei*) [59], annual ryegrass (*Lolium rigidum*) [60], various species of eucalyptus (*Eucalyptus* spp.) [61], Norway spruce (*Picea abies*) [62], and the South American tree *Anadenanthera colubrina* [63]. Bacteria have been detected in different parts of the seed, including the coat, endosperm, and embryonic tissues [37,56,64]. Truyens et al. [7] reviewed studies on seed endophytes, and noted that the bacteria found in seeds tend to belong to specific genera, especially *Bacillus* and *Pseudomonas*, but also *Paenibacillus*, *Micrococcus*, *Staphylococcus*, *Pantoea*, and *Acinetobacter*.

There is evidence that some seed endophytes have beneficial host effects. In ryegrass for example, indigenous endophytes may contribute to releasing seed dormancy though production of cytokinins and interactions between bacterial and plant hormones [60]. Other seed endophytes may promote plant germination and growth under harsh environmental conditions, i.e., by supplying inorganic nutrients through rock weathering and fixing atmospheric nitrogen [59,65]. Additionally, seed endophytes can have anti-fungal properties in vitro [66,67], and inoculation with cadmium (Cd) resistant endophytes isolated from seed protect plants from Cd-toxicity [52]. In another study, removal of rice seed endophytes by surface-sterilization and antibiotic treatments restricted seedling growth relative to control seedlings [49].

Given such beneficial traits, it is conceivable that some plants may have formed mutualisms with bacteria that are vertically transmitted via seed, ensuring continued transmission of beneficial symbionts, similar to what has been described for defensive mutualisms between plants and fungal endophytes [68,69]. To our knowledge, only one obligate vertically transferred plant–bacterium symbiosis has been described. The leaf-nodulating nitrogen-fixing *Burkholderia* symbionts present in the angiosperm genera *Ardisia*, *Pavetta*, *Psychotria*, and *Sericanthe* reside in every vegetative shoot tip and colonize each new leaf [70]. These bacteria are transferred into the floral shoot tip, then the embryo sac of the developing ovule, and eventually, on the epicotyl of the embryo, from which they become enclosed in the shoot tip of the seedling [70]. Despite being obligate and vertically transferred, there is no evidence of co-speciation between hosts and leaf-nodulating *Burkholderia* symbionts, likely due to mixed-mode transmission involving both vertical inheritance and horizontal transfers from the environment, and frequent host switches [71]. It is interesting to note that even in this confirmed vertically transmitted symbiosis, it is still difficult to detect the symbiont in seeds, as the amount of bacterial DNA in seed is low [71].

Presence of bacteria in seed does not mean they originated in the parent, and not all seed-inhabiting bacteria will necessarily colonize seedlings. The structure of seed-associated bacterial communities can tell us something about their origin. Selection by the plant and/or bacterium, as in the case of leaf-nodulating bacteria, should lead to long-standing associations and high similarity in seed communities within a plant species and across related plant species, regardless of environmental factors such as soil type of geographic location (i.e., despite the lack of host–microbe co-speciation, the leaf-nodulating symbionts consistently belong to the genus *Burkholderia*). If, on the other hand, neutral processes dominate in determining the assembly of a seed-associated community, we should expect to see more variation across plant individuals, species, and locations.

The best evidence in support of vertical transfer of endophytes via seed comes from studies that demonstrate overlap in endophyte taxa between seed and seedling, consistent with, but not confirming, vertical transfer of endophytes through seed [61,72,73]. Other studies have reported continuity in the presence of particular strains across generations in rice and maize [67,74], also supporting vertical transfer. And at least in maize, there is some evidence of long-term conservation in the seed endophyte community; seeds of genetically related maize hybrids have been found to host similar bacterial taxa [74], and a study using terminal restriction fragment length polymorphism of 16S rDNA showed presence of the same genera across several genotypes of maize, including its ancestor teosinte [51]. Indirect evidence of vertically transmitted, seed-borne endophytes comes from a study on the invasive Johnsongrass (*Sorgum haplense*), where plants raised aseptically from surface-sterilized seed were shown to acquire nitrogen from a source other than introduced nitrogen, suggesting that diazotrophs had been transferred vertically via seeds [65].

Several recent studies have used high-throughput 16S rRNA sequencing to investigate how the seed microbiome community structure and diversity depends on various factors such as emergence, host genotype, and geography. In *Brassica* spp. and common bean (*Phaseolus vulgaris*), few endophyte operational taxonomic units (OTUs) were conserved across samples, and plant genotype did not seem to be an important driver of the bacterial seed endophyte community, suggesting that neutral processes determine the assembly of seed endophytes in these plant species [75]. The authors also found that bacterial diversity decreased during emergence (defined as apparition of the cotyledon), likely due to an increase in relative abundance of some common seed taxa (e.g., *Pantoea* and *Pseudomonas*), and a decrease or extinction of transient seed colonizers. Truyens et al. [76] investigated the effect of different growth substrates (sand vs. sand/soil mix) on the assembly of the bacterial endophytic community in *Arabidopsis thaliana*, and found that seed and radicle communities were similar to each other, but not to substrate communities, suggesting selection on the part of the plant. However, only a minor part of the seed communities were found in the leaves, which instead appeared to be derived from the non-soil environment, likely the atmosphere or the nutrient solution [76]. While the results from these studies do not rule out vertical transfer of endophytes via seeds, they suggest that most seed endophytes colonize the seed horizontally. However, it is possible that some seed endophytes are occasionally transferred to the next generation; for example, a study of *A. thaliana* suggest that the plant may select seed endophytes based on environmental stressors and pass them on to the next generation [77]. Such intermittent vertical transfer of endophytes may not leave an evolutionary signature on the patterns of host and seed endophyte associations.

As pointed out by Tryuens et al., the presence of identical 16S rRNA sequences across seeds from different genotypes, between seeds of consecutive generations, or between seed and seedling cannot verify vertical transfer until strain-level information is available [7]. In addition, given low amounts of DNA in the seed-transmitted leaf-nodulating symbiosis [71], it may be difficult to definitively prove vertical transfer via DNA sequencing.

Bacteria can colonize seeds horizontally from the external environment via flowers (see Section 3.2.2), fruit, or once seeds are dispersed, though soil or the germination environment (see Section 3.1.1). From studies of pathogenic microbes, we know that developing seeds can be colonized vertically, from the parent plant; microbes can be transmitted or move from vegetative parts of the plants to the developing seed via vascular connections to the endosperm, and can also colonize a seed via pollen [78]. A study by Puente and colleagues provide some evidence of transfer of seed-borne rock-degrading endophytes of cardon cactus [79], consistent with the important role of these endophytes for their host’s establishment on rock surfaces. Endophytic bacteria were observed in in the root cortex and vascular system of seedlings germinated from disinfected seeds, and in surface-disinfected fruit [79]. However, these bacteria were not identified, and may not correspond to the bacteria identified in cactus seeds. Similarly, overlap in endophyte taxa between seed and fruit was observed in grapevine, where mainly *Bacillus* spp. were visualized inside berries between pulp cells and xylem, and along cell walls inside seeds [56].

Another possible route is via the shoot apical meristem (SAM), which consists of undifferentiated cells that give rise to all the post-embryonic aerial organs [80]. Tissues deriving from the SAM, including reproductive organs—and as a consequence, developing seeds—might acquire bacteria residing in the meristem. This route would ensure transfer from the mother plant to the seedling. Shoot tip bacteria are often detected first in tissue culture, since shoot tip meristems or embryos are often used as the starting material for tissue culture [81]. Examples include poplar trees [82], Norway spruce [83], Scots pine (*Pinus sylvestris*) [84,85] papaya [86], banana (*Musa* spp.) [87], sour cherry (*Prunus cerasus*) [88], pineapple (*Ananas comosus*), and orchid (*Oncidium flexuosum*) [89]. Although bacterial endophytes generally are reported to reside in intercellular spaces, many tissue culture or shoot tip endophytes have been observed inside plant cells [90,91,92]. In Scots pine, in situ hybridization was used to detect endophytes in intact buds, where they were found to reside inside cells of scale primordia, meristems, and around the resin ducts buds [84]. rRNA abundance of these endophytes was quantified and found to be highest prior to growth or differentiation of the bud [93].

### 2.2. Vertical Transfer via Pollen

As mentioned above, one possible way that endophytes could get into seed is via the male gametes. Bacteria have been identified both inside and on the surface of pollen of different plant species [58,94,95,96,97,98]. Pollen grains are exposed to the outer environment, and could be colonized horizontally from the atmosphere, or via pollinators or other animals. If the bacteria in or on pollen originate from within the plant, their transfer to seed and seedling would constitute vertical transmission. The isolation of the endophyte *Enterobacter cloacae* from surface-sterilized pollen of the Mediterranean pines Aleppo pine (*Pinus halepensis*), stone pine (*Pinus pinea*), and Turkish pine (*Pinus brutia*) suggests an origin within the parent plant, and the isolation of the same bacterial species from fertilized *P. brutia* ovules [95] may indicate vertical transfer of *Enterobacter* spp. in pines via pollen.

A recent study described the abundance, community structure, diversity, and colonization pattern of bacteria associated with two wind-pollinated and two insect-pollinated species of plant: birch (*Betula pendula*), rye (*Secale cereal*), rape (*Brassica napus*), and autumn crocus (*Colchicum autumnale*) [94]. Bacteria were abundant (10^6^–10^9^ cultivatable bacteria per gram of pollen), and occurred on the outer surface as single cells, clusters, or as thin biofilms. The communities differed significantly between plant species, potentially as a result of the species-specific difference in pollen structure, nutritional composition, or antimicrobial peptides on the pollen coat [94,99]. Bacterial communities from insect-pollinated species were more similar to each other than to bacterial communities from wind-pollinated species, suggesting perhaps an influence of pollinators on pollen bacterial community composition (see Section 3.2.2). In wind-pollinated species, pollen itself may serve as a vector for horizontal transmission of the plant microbiome (discussed in Section 3.2.2).

## 3. Horizontal Transmission

### 3.1. Colonization of Seed and Root via Soil

The microbial seed bank that is soil hosts a plethora of microorganisms capable of surviving for thousands of years [100]. Soil is considered the most important source of inoculum for endophytes [3,36], serving as a reservoir for both below- and aboveground plant microbiota. In a recent study of the epi- and endophytic bacteria associated with grapevine (*Vitis vinifera*), the communities associated with leaves, flowers, and grapes shared a greater proportion of taxa with soil communities than with each other, suggesting a soil origin for above- as well as belowground communities [101]. Over 100 years ago, Victor Gallipe posited that microorganisms from the soil penetrate plants [102], and some years later, Lorenz Hiltner discovered a rich zone of bacteria surrounding plant roots, and coined the term “rhizosphere” [103]. For soil-borne bacteria to establish as endophytes via the roots, they must first pass through the rhizosphere. Therefore, some of the same biotic and abiotic factors that influence the structure and assembly of the rhizosphere microbiome likely influence the communities of microorganisms transmitted to the root interior.

#### 3.1.1. Endophytic Colonization of the Spermosphere

Colonization via the soil begins in the spermosphere, the short-lived and microbiologically dynamic zone surrounding a germinating seed, where bacteria can have beneficial effects on germination [104,105]. Seeds exudate carbon in the form of sugars, proteins, and fatty acids (reviewed in Nelson [104]). A likely energy source for microbes, these compounds have the potential to shape the bacterial composition of the soil surrounding the seed [106,107,108]. These early relationships, potentially selected by the plant, may be important for setting up beneficial interactions [105]. The finding that microbes of the spermosphere differ from these detected in the rhizosphere indicate that seeds select for specific microorganisms, which can colonize the sowed seed within a few hours [104].

#### 3.1.2. Colonization of the Root Endosphere via the Rhizosphere

The rhizosphere, or the millimeters of soil that directly surrounds the root, is teeming with bacteria that promote host growth and development, assist with nutrient acquisition, protect against pathogens, induce systemic resistance, and support growth under abiotic stress, such as toxic pollutants or drought [109,110,111,112,113]. Plants grown hundreds of kilometers apart assemble similar rhizosphere communities [114], illustrating the plant’s capacity to determine the composition of the rhizosphere microbiome, also called the “rhizosphere effect” [115]. Rhizosphere community composition depends on plant genotype [116,117], but the strength of the rhizosphere effect differs between plant species [118], and in some cases, soil type can dominate over plant genotype in structuring rhizosphere bacterial communities [30,31].

Plants secrete rhizodeposits and exudates that attract soil microbes and increase rhizosphere populations near plant roots [115,118,119,120], investing a significant amount of fixed carbon and nitrogen to recruit and modulate communities of bacteria. Additionally, plant roots modulate their microbiota by influencing the soil pH, soil structure, and oxygen availability of the surrounding soil [121,122]. Plants use these selection capabilities at the time of establishment, throughout the course of their life, and in response to other organisms, climate variables, soil microbial composition, and to specifically recruit microbes that facilitate adaptation to stress [115,122]. For example, plants that are subject to pathogen or insect attack can recruit protective bacteria and enhance microbial activity to suppress pathogens in the rhizosphere [123]. The bacteria themselves are also actively involved in the colonization process, using traits such as motility, chemotaxis and quorum-sensing to aggregate near the roots of plants or compete with neighboring bacteria [124,125,126,127,128,129]. The mechanisms behind plant-mediated modulation of the rhizosphere and root endosphere microbiomes are currently being been unraveled; it has been shown that defense hormone signaling selects specific bacterial families for colonization from the available microbial communities [130], and shapes the root microbiota during plant nutrient stress [131].

Recent evidence suggests that the rhizosphere has direct influence over the bacteria that colonize the rhizoplane (the root surface) and subsequently the interior, as endophytes. In a study of the rice root microbiome, next generation sequencing of the 16S rRNA gene was used to distinguish three distinct, but overlapping bacterial communities in the rhizosphere, rhizoplane, and endosphere at high resolution and depth [34]. Only a subset of rhizosphere bacteria were found at the rhizoplane, suggesting that bacterial colonization of rice root surfaces is an active process, where plants select for certain microbial consortia, or where some bacteria are better at invading the root surface, for example, by forming biofilms [34]. Additional depletion of rhizoplane taxa from the endosphere suggested a further selective step for entering the root interior, implying that although binding at the rhizoplane may be a necessary prerequisite, it is not sufficient for root entry. Each of these steps likely involves root exudates and molecular signals from the plant. Transplantation of seedling roots from sterile media to field soil demonstrated rapid microbial penetrance into the endosphere, starting within a day after transplantation, and approaching steady state within two weeks [34].

A two-step model for endophytic entry via the rhizoplane is supported by earlier microscopy studies. For example, inoculation of *V. vinifera* with *Burkholderia* sp. strain PsJN tagged with green fluorescent protein (*gfp*), demonstrated chronological colonization starting on root surfaces, then in root internal tissues, and finally, in xylem vessels of internodes and leaves [26], and in a follow-up study, inside young berries [132]. The bacterium was found to secrete cell wall-degrading endoglucanase and endopolygalacturonase, potentially explaining penetration into the root endosphere [26]. In several studies, bacteria have been localized to lateral root emergence sites and root tips, suggesting that entry is facilitated by cracks in the root, and that bacteria enter through root tips [26,133,134]. Reports of root inoculants subsequently localized in the xylem vascular system and aerial plant parts demonstrate that all plant parts can be colonized by soil bacteria [26,40,132,133,135]. However, not all root bacteria colonize the rest of the plant; some specialize in roots, or even different part of roots (fine, secondary, primary), as demonstrated by a study of tobacco (*Nicotiana tabacum*) [136].

### 3.2. Entry into Aerial Tissues

In addition to entering roots via soil and moving through the xylem vessels, endophytes can enter aerial tissues via above-ground surfaces, including stem, leaves, flowers (anthosphere), and fruits (carposphere). Potential bacterial source environments include the atmosphere, rain, soil, or pollinators or other insects. There is evidence to suggest that bacteria enter leaves and stem via stomata. The stomatal route of entry has been studied for plant pathogens, but less so for endophytes. However, overlap in community composition between leaf surface and interior [137] suggests that the leaf surface represents the initial phase of colonization for some foliar endophytes. Therefore, in order to understand the endophytic colonization aerial plant parts, we need to consider the transmission and dispersal routes of plant surface-dwelling bacteria.

#### 3.2.1. Aerial Dispersal of the Plant Microbiome

The aerial surface of plants, termed the phyllosphere, is considered one of the most prevalent microbial habitats on the planet [138,139]. Plant epiphytes can colonize the plant from within; for example, it has been show that seed-borne bacteria inoculate plant surfaces via the germinating seedling [75]. However, many phyllosphere bacteria are likely deposited via bioaerosols, which are minute particles that include bacteria, fungi, viruses, or pollen, released from terrestrial and marine environments into the atmosphere [140]. Bacteria are particularly abundant in the atmosphere, with concentrations ranging from 10^4^ to 10^6^ cells/m^3^ [141]. Bioaerosols can contain single cells or aggregates of bacteria, bacterial spores, or bacteria aggregated with dust particles, small plant debris, or pollen [140]. Because many bioaerosol bacteria are viable and metabolically active in the atmosphere [142,143,144,145], bioaerosol formation is considered a major mechanism of bacterial dispersal and migration on global and continental scales [146,147,148]. Airborne dust, in particular, is considered a major mode of transport for microbes; dust storms can transport microscopic particles thousands of kilometers away from the source [149,150,151]. For example, characterization of the microbial communities in airborne dust deposition demonstrates that dust storms can transport viable bacteria from Saharan soils to high altitude areas in Europe, and that sporulation is not necessary [152,153].

Bioaerosols have been studied for their ability to disperse plant- and animal pathogens over long distances [154,155], but less for their ability to disperse beneficial plant microbiomes. Plant canopies dominate Earth’s land surface, and are considered the major source of particles in the atmosphere. Early attempts to quantify the viable bacteria in the atmosphere found much higher concentrations of bacteria over plant canopies than over soil [156], suggesting that plant canopies constitute a major source of airborne bacteria. More recent characterization of airborne communities over agricultural fields, suburban areas, and forests, point to soil and plant surfaces as the origin for a portion of the bacteria in the near-surface atmosphere [157]. Similarly, fungal communities in the atmosphere over the Amazon rainforest were found to be most similar to communities found in tropical soils and leaf surfaces [158]. A recent study demonstrated that a single drop of water splashing on the ground can aerosolize thousands of bacteria [159]. Observations of aerial dispersal of epiphytes from bean plants suggests a pattern of high bacterial upward flux from dry leaves on sunny days, and a high downward flux and lateral movement of bacteria on rainy days [160]. Studies of plant pathogens demonstrated that dispersal is facilitated by rain and sprinkler irrigation [161,162].

Once aerosolized from canopies, bacteria can disperse laterally to surfaces of different leaves, individuals, or species of plants [163,164], or upwards by air currents, where they can be transported by wind much faster and wider than other mechanisms, such as diffusion through soil, before being deposited again by precipitation or dry deposition [147]. In contrast, dispersion of soil bacteria is limited enough to create regional endemism [165,166].

It is not known what fraction of the endophytic microbiome is dispersed via the atmosphere, if any, but overlap between endophytic and airborne communities suggests that it is a possibility to consider. For example, the Alphaproteobacterial order Rhodospirillales dominated the viable airborne bacterial community above an Oregon mountaintop [145], and the community deposited on subalpine snow in California [167]. Interestingly, the Rhodospirillales have been found consistently in the foliar endophytic community of multiple species of subalpine conifers in California and Colorado [15,168,169], potentially suggesting interactions between the air and conifer canopy microbiome in subalpine regions.

#### 3.2.2. Endophytic Leaf Colonization via Stomata

Unlike epiphytic fungi, bacteria colonizing the surface of leaves are not known to penetrate the leaf cuticle [170,171,172], but studies on bacterial pathogens (and to some extent, endophytes) suggest that they may use openings in the plant epidermis, including the stomata (openings in the aerial part of plants that allow and control gas exchange and water transpiration between the plant interior and the atmosphere), lenticels (raised pores in the stem of woody plants that also allow gas exchange), and hydrathodes (water-secreting pores usually present near the leaf margin). Bacterial plant pathogens can enter through all these openings, but the stomata dominate in number and are considered the main route of entry to the interior of plant aboveground parts [170,173,174]. The stomata are formed by a pair of guard cells that control the stomatal pore in response to light intensity, carbon dioxide concentration, and relative humidity. In addition, the stomata represent a first line of defense against bacterial pathogens, as the guard cells can sense microbe-associated molecular patterns (MAMPs) and close the stomatal pore in response, in a process mediated by phytohormones [175]. In return, some pathogens have evolved mechanisms to counter the stomatal defense and open the stomatal pore via virulence factors that include phytotoxins that prevent MAMP-triggered stomatal closure [176,177,178,179], and effectors secreted by type III secretion systems that either prevent closure or induce opening of the pore [180,181]. Stomatal defense has also been shown against the human pathogen *Escherichia coli* O157:H7, suggesting that plants actively police not just plant pathogens against entering the stomata. Another human pathogen, *Salmonella enterica* serovar Typhimurium SL1344S, uses chemotaxis to migrate towards stomata, where it causes a transient stomatal closure, but is able to colonize the apoplast, suggesting in can avoid or subvert plant immunity [182,183]. These results suggest that there is variation in stomatal response to phyllosphere bacteria depending on the natural variation of bacterial MAMPs [184].

The fact that plants recognize and exclude particular bacteria from entering their stomata, and that the bacteria develop mechanisms to counter such responses, raises the question if commensal or beneficial phyllosphere bacteria are allowed—and possibly recruited—to enter the stomata, and to colonize the above ground parts as endophytes. There is some evidence that bacteria enter stomata without harming the plant (i.e., as endophytes). The growth-promoting nitrogen-fixing endophyte, *Herbaspirillum seropedicae* has been shown to enter the stomata of pineapple (*Ananas comosus*) [185]. Aggregates of bacteria were found on trichomes, epidermal cell wall junctions, and in the vicinity of stomatal complexes, followed by penetration through the stomata into the substomatal chamber, and colonization of the intercellular spaces of the leaf mesophyll. A similar observation of clusters of bacteria near stomata was made while imaging native bacteria in maize leaves [186]. The presence of bacteria in the upper side of leaves was suggested as indirect evidence for colonization of the host after stomatal penetration. The vanilla orchid (*Vanilla phaeantha*) endophyte *Bacillus amyloliquefaciens* was found in shoot meristems and stomatal areas of stems and leaves, and inside guard cells and other epidermal cells in the surrogate host *Amaranthus caudatus* [187]. Further support for stomatal colonization by beneficial bacteria comes from a study of the diazotroph *Azospirillum brasiliense*. When applied to maize and wheat via leaf spray inoculation (with controls for entry via soil), the bacteria did not survive in the phyllosphere but were found to have colonized the interior of leaves and stem, most likely via the stomata [188].

#### 3.2.3. Floral Transmission of Bacterial Endophytes

The surfaces of different floral organs host diverse communities of bacteria [2]. Culture-dependent and independent studies have described bacteria in nectar [189,190,191,192], petals [58,192], pistil [58,193], and as mentioned previously, in pollen [95,96] and fruit [64,132,194]. The bacteria that colonize flower surfaces can originate from the same sources as the bacteria that colonize leaf surfaces (i.e., air, dust, wind, rain splash, and surrounding plants and soil, or from pollinators and other insect visiting flowers [195]). In a study of the microbial communities associated with apple blossoms, wind was correlated with temporal community patterns, and likely acts as an agent of dispersal to and from flowers [196]. To our knowledge, endophytic colonization of flowers via the environment has not been described, but we know from studies of the apple and pear fire blight agent *Erwinia amylovora*, which primarily infects flowers [197,198,199] that environmentally derived bacteria can penetrate floral tissue. Epiphytic *E. amylovora* infections develop on the stigma or the hypanthium (where nectar is secreted), and the bacteria enter the plant though the nectarthodes [198,200,201].

Petals also have stomata, through which epiphytes could potentially gain entrance, but to our knowledge, this has not been reported. However, it is known that bacteria that are horizontally acquired via flowers can enter developing seeds, and thereby colonize the offspring. Mitter and collaborators [37] introduced endophytes into seeds of maize, pepper, and soybean by spraying a bacterial inoculant directly on the flower of the mother plant. Afterwards, they were able to detect the used bacterial strain in the cotyledons of the embryo/seedling, and showed that the bacteria are able to proliferate and colonize the root and stem of the offspring.

Flowers potentially provide the plants with two predictable transmission routes for the aerial tissue microbiome: insect vectors, and in the case of wind-pollinated species, the pollen itself. Besides pollinators, flowers are visited by predators in search of prey [202], herbivores that feed on floral tissue [203], and insects looking for a mate [204]. Indeed, flowers are hotspots of invertebrate biodiversity, supporting densities that are ten to ten thousand times greater than on the nearby foliage [205]. Surveys of flower and pollinator microbiomes suggest that pollinator visitation may influence floral microbiomes and that flowers serve as hubs of transmission of pollinator bacteria [189,206,207].

As discussed above, pollen of both insect-pollinated and wind-pollinated plants are host to a diverse community of bacteria. Since pollen grains are released into the environment in vast quantities—Molina et al. [208] reported the release of up to 500,000 million grains for an individual tree—they may be an efficient vector not only for vertical transmission of endophytes, but also for canopy-to-canopy horizontal transmission of plant microbiomes. Pollen-mediated dispersal of microbes would not require viable pollen and fertilization, and could occur over considerable distances. Transport of tree pollen has been documented at 600 km for viable pollen [209] and 3000 km for potentially viable pollen [210]. There is some evidence to suggest that fungal plant pathogens use the pollen transmission route [211].

#### 3.2.4. Endophyte Transmission by Plant-Feeding Insects

Sap-feeding insects, such as leafhoppers, planthoppers, and psyllids in the insect order Hemiptera, can be vectors of plant disease, including viruses and bacteria, such as *Phytoplasma* (an obligate plant pathogens) and *Xylella* [212,213,214]. These insects have piercing–sucking mouthparts that enable them to puncture phloem or xylem cells and suck out the contents, sometimes transmitting plant pathogens in the process. Interestingly, *Cardinium*, an intracellular symbiont of many sap-feeding insects, can be horizontally transmitted between different phloem sap-feeding insect species through plants [178], and *Wolbachia* associated with the phloem-feeding whitefly (*Bemisia tabaci*), can be horizontally transmitted via multiple species of plants, where it has been visualized in situ in phloem vessels and “reservoir” spherules along the phloem [215]. These results suggest that *Cardinium* and *Wolbachia* can reside in the plant as endophytes, at least temporarily, and that non-pathogens can be transmitted between plants via sap-feeders. A recent study of the American grapevine leafhopper *Scaphoideus titanus*, a phloem-feeder, investigated the potential transmission of entire communities endophytes [216]. In the experiment, insects were allowed to first feed on source plants raised under natural conditions and hosting typical microbial communities, and then on axenically micropropagated grapevine plantlets. The experiment was repeated four times, revealing that sink plants were colonized by a microbiome that was very similar to that in the source plant, and suggesting that the majority, if not all endophytes were transmitted this way. Interestingly, the endophytic community was found not just in the stems where the insects fed, but throughout the sink plant, including in the roots [216]. Thus, sap-feeding insect are potential vectors of the beneficial or commensal plant microbiome within and between plant species. For example, beneficial phloem endophytes, as the *Bacillus pumilus* strain isolated from lodgepole pine (*Pinus ponderosa*) and found to be antagonistic against a fungal symbiont of the mountain pine beetle (*Dendroctonus ponderosae*) [17], could be transmitted this way. On the other hand, many sap-feeders appear to host relatively depauperate bacterial communities [214]. Clearly, more research is needed to determine if sap-feeders are significant vectors of plant microbiomes.

## 4. Conclusions

Studies from a range of plant species demonstrate the importance of the environment on structuring the endophytic bacterial microbiome, suggesting that it is predominantly horizontally transferred. In addition, many bacterial endophytes appear to be generalists, both in terms of the plant organ they colonize and in terms of host species. Obligate relationships between bacteria and plants may be rare, as few have been identified to date. Evidence of vertical transmission is generally indirect, and transfer of vertically transmitted endophytes from seed to reproductive organs within the plant needs to be demonstrated. There is evidence that some of the bacteria identified in seeds undergo mixed-mode transmission. The best studied and understood transmission route for bacterial endophytes is colonization of roots via the soil and rhizoplane. The role of aerial transmission and uptake via stomata is less clear and merits further study, as does the potential use of pollinators and other insects as vectors for endophytes. In addition, the relative importance of transmission to aerial parts in shaping the plant microbiome is unknown. A predominately horizontally transmitted and generalist microbiome fits well with the emerging view that recruitment and modulation of microbiome is a plant strategy to adapt to a changing environment. On the other hand, it is possible that some plants—like the rock weathering cardon cactus—require specific microbes for growth in their natural habitat, and that seed-borne transmission has evolved as a consequence.
